# Development of a Clinical Simulation Video to Evaluate Multiple Domains of Clinical Competence: Cross-Sectional Study

**DOI:** 10.2196/54401

**Published:** 2024-02-29

**Authors:** Kiyoshi Shikino, Yuji Nishizaki, Sho Fukui, Daiki Yokokawa, Yu Yamamoto, Hiroyuki Kobayashi, Taro Shimizu, Yasuharu Tokuda

**Affiliations:** 1 Department of Community-Oriented Medical Education Chiba University Graduate School of Medicine Chiba Japan; 2 Department of General Medicine Chiba University Hospital Chiba Japan; 3 Division of Medical Education Juntendo University School of Medicine Tokyo Japan; 4 Department of Emergency and General Medicine Kyorin University Tokyo Japan; 5 Division of General Medicine Center for Community Medicine Jichi Medical University Tochigi Japan; 6 Department of Internal Medicine Mito Kyodo General Hospital Tsukuba Japan; 7 Department of Diagnostic and Generalist Medicine Dokkyo Medical University Hospital Tochigi Japan; 8 Muribushi Okinawa Center for Teaching Hospitals Okinawa Japan; 9 Tokyo Foundation for Policy Research Tokyo Japan

**Keywords:** discrimination index, General Medicine In-Training Examination, clinical simulation video, postgraduate medical education, video, videos, training, examination, examinations, medical education, resident, residents, postgraduate, postgraduates, simulation, simulations, diagnosis, diagnoses, diagnose, general medicine, general practice, general practitioner, skill, skills

## Abstract

**Background:**

Medical students in Japan undergo a 2-year postgraduate residency program to acquire clinical knowledge and general medical skills. The General Medicine In-Training Examination (GM-ITE) assesses postgraduate residents’ clinical knowledge. A clinical simulation video (CSV) may assess learners’ interpersonal abilities.

**Objective:**

This study aimed to evaluate the relationship between GM-ITE scores and resident physicians’ diagnostic skills by having them watch a CSV and to explore resident physicians’ perceptions of the CSV’s realism, educational value, and impact on their motivation to learn.

**Methods:**

The participants included 56 postgraduate medical residents who took the GM-ITE between January 21 and January 28, 2021; watched the CSV; and then provided a diagnosis. The CSV and GM-ITE scores were compared, and the validity of the simulations was examined using discrimination indices, wherein ≥0.20 indicated high discriminatory power and >0.40 indicated a very good measure of the subject’s qualifications. Additionally, we administered an anonymous questionnaire to ascertain participants’ views on the realism and educational value of the CSV and its impact on their motivation to learn.

**Results:**

Of the 56 participants, 6 (11%) provided the correct diagnosis, and all were from the second postgraduate year. All domains indicated high discriminatory power. The (anonymous) follow-up responses indicated that the CSV format was more suitable than the conventional GM-ITE for assessing clinical competence. The anonymous survey revealed that 12 (52%) participants found the CSV format more suitable than the GM-ITE for assessing clinical competence, 18 (78%) affirmed the realism of the video simulation, and 17 (74%) indicated that the experience increased their motivation to learn.

**Conclusions:**

The findings indicated that CSV modules simulating real-world clinical examinations were successful in assessing examinees’ clinical competence across multiple domains. The study demonstrated that the CSV not only augmented the assessment of diagnostic skills but also positively impacted learners’ motivation, suggesting a multifaceted role for simulation in medical education.

## Introduction

Japan’s medical schools follow a 6-year curriculum comprising 4 years of preclinical and 2 years of clinical education, after which they enter a 2-year postgraduate residency program as “postgraduate residents” or simply “residents” [1-3]. This residency enables new doctors to acquire and practice basic clinical knowledge, problem-solving, general medical and communication skills, and a professional attitude. All residents receive supervised training as they rotate through 7 specialties over the 2 years, including internal medicine, surgery, pediatrics, obstetrics and gynecology, psychiatry, emergency medicine, and community medicine. Most residents then enter specialty-based residency training.

In 2011, the nonprofit Japan Institute for Advancement of Medical Education Program (JAMEP) developed the General Medicine In-Training Examination (GM-ITE), an in-training examination for assessing the clinical knowledge of residents, similar to the US Internal Medicine Residency Examination [4]. The purpose of the GM-ITE is to elicit practical feedback on the training programs aimed at identifying improvement areas using an objective and reliable assessment of residents’ clinical knowledge [5].

The traditional assessment of clinical competencies through multiple-choice questions (MCQs), while valuable, may not encompass the full scope of a clinician’s diagnostic process in real-world practice [6]. In clinical settings, physicians must navigate through complex problem-solving and decision-making processes, often divided into domains such as leading or working diagnosis, management and treatment, hypothesis generation, problem representation, diagnostic justification, and information gathering [7]. Video simulation, as an assessment tool, can capture these nuances by providing contextualized real-world scenarios where residents must apply their knowledge dynamically, as they would in actual patient interactions [8].

Designed by a committee of experienced attending physicians organized by the JAMEP, the 2-hour GM-ITE comprises 80 MCQs covering multiple domains [9]. The scores range from 0 to 80, with higher scores indicating better performance and knowledge of internal medicine. The content and validity of each question undergo review by JAMEP’s question-development committee comprising experienced physicians from various fields, an independent peer-review committee, and examination-analysis experts [10]. The GM-ITE is not used as a pass or fail test for training advancement but only as a source of education feedback. The test is strictly voluntary, and approximately one-third of residents take the examination each year (7669 in the 2020 academic year, 6869 in the 2019 academic year, 6133 in the 2018 academic year, 5593 in the 2017 academic year, and 4568 in the 2016 academic year) [11,12].

An assessment of the validity of the GM-ITE [10] revealed a strong positive correlation between GM-ITE scores and scores on the Professional and Linguistic Assessments Board test, Part 1, designed to assess the depth of medical knowledge and levels of medical and communication skills [13]. In validity testing, the discrimination index (DI) indicates how well the item differentiates between students of high and low aptitude, that is, whether high-aptitude students performed better, worse, or the same as low-aptitude students [14]. Therefore, an item with a high DI is more effective in identifying respondents with adequate knowledge than an item with a low DI. The GM-ITE has indicated better discriminative power than the Professional and Linguistic Assessments Board test, Part 1 examination [10].

The JAMEP based the content of the GM-ITE on the clinical training objectives presented by Japan’s Ministry of Health, Labour and Welfare [13], which requires residents to master skills related to professionalism, physical examination and clinical procedures, and the diagnosis and treatment of common diseases. The GM-ITE shows evidence of generalization by covering 4 categories, including medical interview or professionalism (MP), clinical diagnosis (CD) consisting of symptomatology and clinical reasoning, physical examination or procedure (PP), and disease knowledge (DK). However, the relatively small number of questions in the GM-ITE provides evidence of low generalization.

Given the large number of residents taking the GM-ITE each year, using MCQs seems both expedient and appropriate when considering the viability and sustainability of the GM-ITE. However, a 2-hour test comprising only MCQs may not adequately assess the situational variations affecting clinical performance or competence in multiple domains. Therefore, this study developed a clinical simulation video (CSV) named “innovative examination” for the GM-ITE to assess residents’ clinical competency in a real-world setting using two components: (1) a high-quality CSV showing a medical interview and physical examinations with a patient and family in an emergency room and (2) follow-up questions for the residents to provide their diagnosis and recommendations. The study then evaluated the relationship between the participants’ GM-ITE and CSV innovative examination test scores by comparing their discriminative ability in each assessment domain. Therefore, this study aimed to evaluate the relationship between GM-ITE scores and resident physicians’ diagnostic skills by having them watch a CSV and to explore resident physicians’ perceptions of the CSV’s realism, educational value, and impact on their motivation to learn.

## Methods

### Study Design

We conducted a multicenter cross-sectional observational study in Japan.

### Study Participants

The study extended an invitation to all 8526 resident physicians who took the GM-ITE in the 2021 academic year (January 21-28, 2021) to voluntarily participate in the innovative examination, and 56 residents—23 from postgraduate year (PGY) 2 and 33 from PGY 1—agreed and participated. These individuals were selected from the entire cohort of residents who took the GM-ITE. Owing to the exploratory nature of this study and the extensive distribution of the questionnaire to all eligible resident physicians, no formal sample size calculation or power analysis was performed.

### Procedures

#### Innovative Examination Using High-Quality Patient-Simulated Video

In this study, we wrote a script depicting a simulated clinical interaction. The approximately 5-minute video (“innovative examination”), shot from a resident’s point of view, depicts a newly arrived patient and his family at an emergency room (Multimedia Appendix 1). The resident conducts a medical interview and examination, asking and answering questions, while the camera records the patient’s and family members’ verbal and nonverbal responses. Professional actors coached by the medical supervisors played the roles effectively. A professional television production company shot the video and added effects (eg, heart sounds). In total, 3 of the authors (KS, YN, and SF) and 3 JAMEP medical supervisors oversaw the video production. The study participants watched the video immediately after completing the GM-ITE. Next, they answered the CSV innovative examination questions described below.

#### Extended Matching Questions

We used extended matching questions that listed the patient’s symptoms to obtain up to 3 pertinent positive findings that contributed to the diagnosis (Q1 and Q2 in Textbox 1).

**Clinical simulation video (CSV) innovative examination questions.**

- Q1. Which 3 physical findings would you expect to be positive in this patient? Please choose 3 of the following:
  Pallor of the eyelid conjunctiva
  Pupil irregularity
  Angry external jugular vein
  Cervical vascular murmur
  Thyroid gland enlargement
  “Fixed” splitting of the second heart tone
  Loud P2
  Systolic murmur
  Diastolic murmur
  Torsion sound at the base of the lung
  Tender points in the abdomen
  Fresh blood in stool on rectal examination
  Barre sign positive
  Muscle stiffness
  Loss of tendon reflexes
- Q2. Please state the most likely diagnosis for this patient (free text).
- Q3. Following the SBAR (situation, background, assessment, and recommendation) format, please prepare a patient handoff record for the internal medicine physician in charge of admission.
  - Q3-1. Situation (free text, 100 words maximum)
  - Q3-2. Background (free text, 100 words maximum)
  - Q3-3. Assessment (free text, 100 words maximum)
  - Q3-4. Recommendation (free text, 100 words maximum)
- Q4-1. Do you think the simulated patient-examination video was better suited to assessing your clinical competence than the traditional all-text format?
- Q4-2. Was the video simulation realistic enough for you to assess the patient?
- Q4-3. Did this experience increase your motivation to learn?

#### Modified Essay Questions

The third question required brief free-form answers (Q3 in Textbox 1).

#### Anonymous Posttest Questionnaire

After the participants completed Q1-Q3, we asked them to answer a fourth question (anonymously) to briefly describe (in writing) their experiences with the CSV innovative examination (Q4 in Textbox 1). Only 23 (41%) of the 56 participants chose to answer Q4.

#### Measurements

The GM-ITE uses a methodology similar to the US Internal Medicine Residency Examination [4,15,16]. The 80 questions cover 4 main categories: MP (8 questions), CD (18 questions), PP (18 questions), and DK (36 questions). We examined the validity of the GM-ITE questions using the DI *φ* as defined by equation 1 [17]:



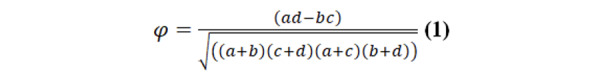



where *a* is the number of correct answers in the top 25th percentile, *b* is the number of incorrect answers in the top 25th percentile, *c* is the number of correct answers in the bottom 25th percentile, and *d* is the number of incorrect answers in the bottom 25th percentile. The range of *φ* is –1≤*φ*≤1. Questions are considered unreliable if this index is below 0. A DI of ≥0.20 would indicate that the question has high discriminatory power, and a DI of ≥0.40 would indicate that the question is a very good measure of the subject’s qualifications.

### Statistical Analyses

We conducted these analyses using SPSS Statistics for Windows (version 26.0; IBM Corp), following the Strengthening the Reporting of Observational Studies in Epidemiology guidelines. Two authors (KS and SF) independently assessed the answers and then discussed, identified, and agreed on them. We measured the interrater reliability with the κ coefficient (0.8-1.0=almost perfect, 0.6-0.8=substantial, 0.4-0.6=moderate, and 0.2-0.4=fair) [18]. The Angoff method was used to define the cutoff for the DI calculation [19].

### Ethical Considerations

This research was conducted in accordance with ethical standards and the principles of the Declaration of Helsinki. The ethics review board of the JAMEP, Tokyo, Japan, approved the study protocol (21-10). All participants read and signed the informed consent document before participating in the study. To ensure confidentiality, all participant data were anonymized prior to analysis. No compensation was provided to the participants for their involvement in this study. Informed consent was obtained from all participants for publication of identifying information in an online open-access publication. In accordance with ethical standards and journal policy, we have obtained explicit informed consent from all actors appearing in the video material associated with this study. The actors have acknowledged and agreed that the video will be published as part of the study’s material.

## Results

A total of 8526 residents from 642 teaching hospitals in Japan took the GM-ITE in the 2021 academic year. Among these, 56 (23 PGY 2 and 33 PGY 1) residents also agreed to take the CSV innovative examination. The mean GM-ITE score of all 56 participants was 47.8 (SD 8.2). A DI revealed that several items had discrimination indices exceeding 0.2 (Table 1).

A total of 6 (11%) out of 56 participants answered Q2 correctly, and all the correct answers came from PGY 2 residents. The DI for the entire CSV innovative examination portion of the GM-ITE indicated high discriminatory power in all domains.

**Figure 1:**
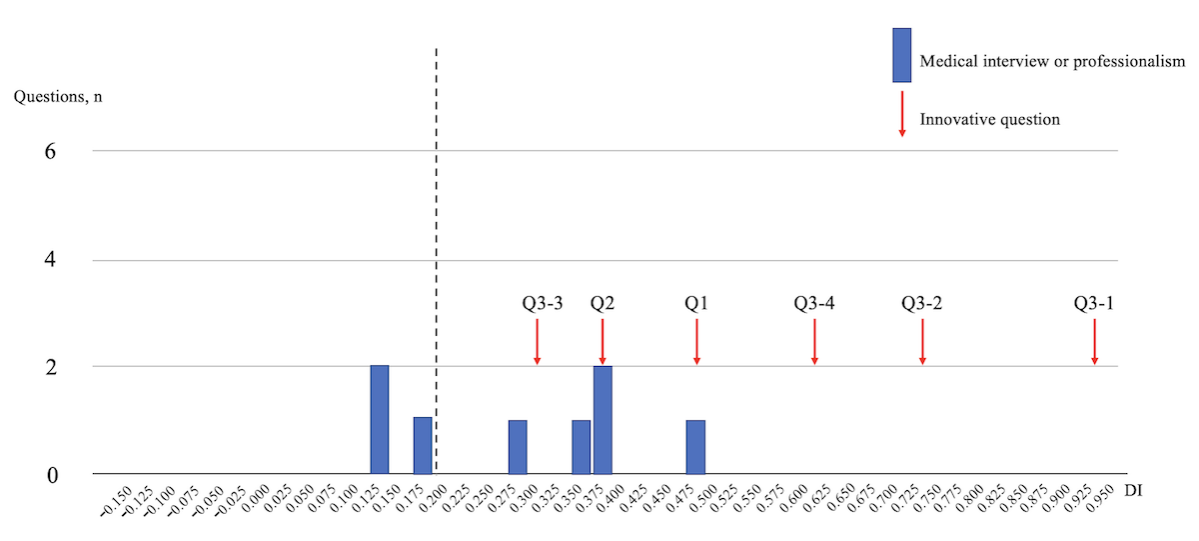
DIs of the examination scores of the General Medicine In-Training Examination: medical interview or professionalism (8 questions). DI: discrimination index; Q: question.

Figure 1 shows the DI for the MP (8 questions) domain, with 6 innovative questions scoring a DI of ≥0.20, indicating its robustness in differentiating examinee proficiency.

**Figure 2:**
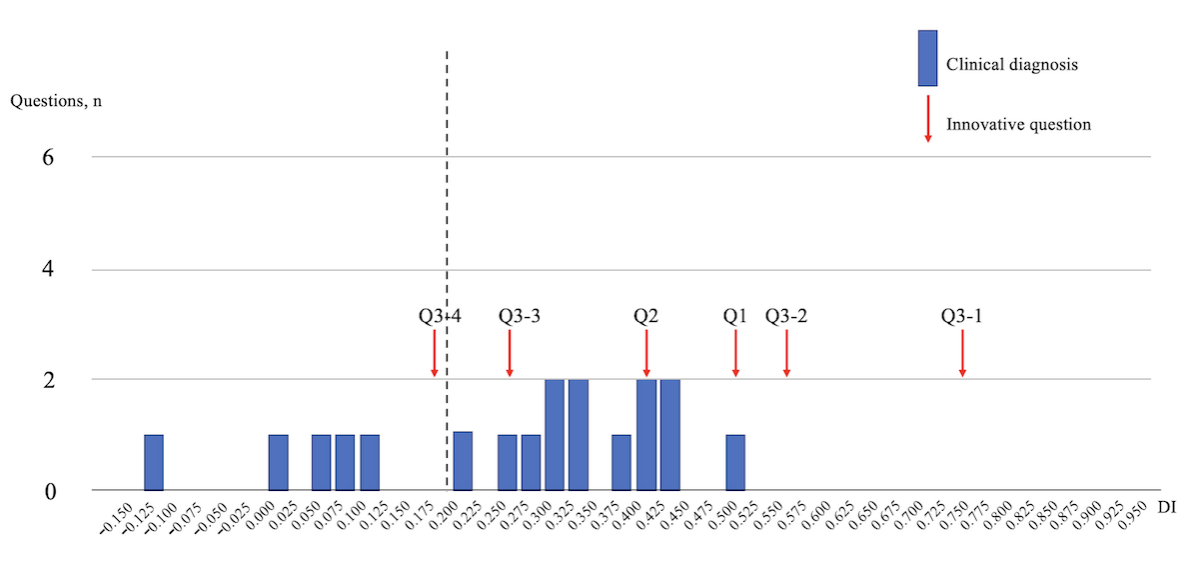
DIs of the examination scores of the General Medicine In-Training Examination: clinical diagnosis (18 questions). DI: discrimination index; Q: question.

Figure 2 focuses on the CD (18 questions) domain, with 5 innovative questions achieving a DI of ≥0.20, which is indicative of its strong discriminatory capability among examinees.

**Figure 3:**
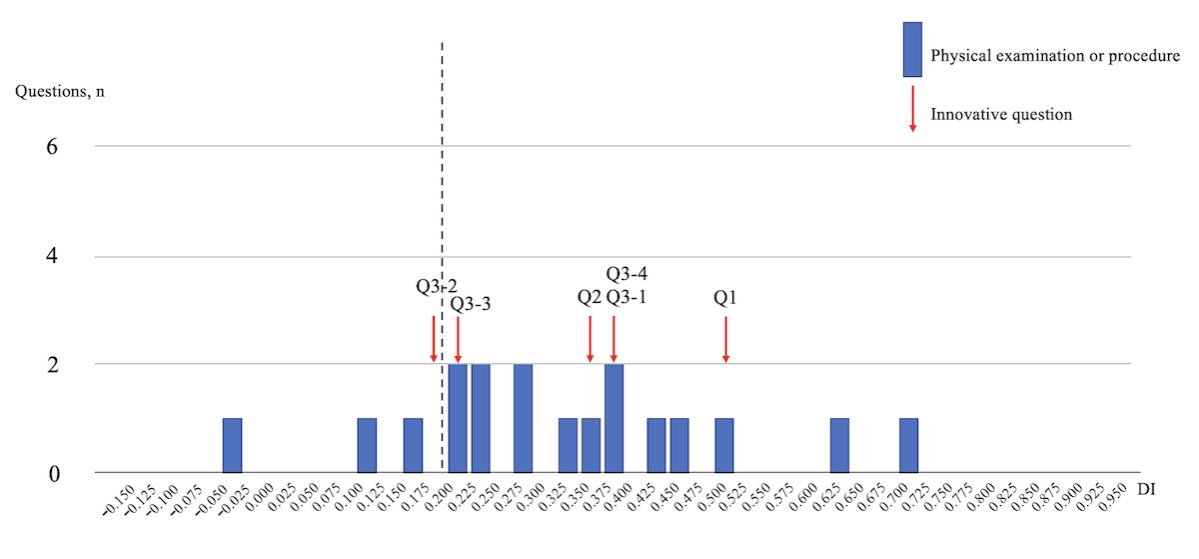
DIs of the examination scores of the General Medicine In-Training Examination: physical examination or procedure (18 questions). DI: discrimination index; Q: question.

In Figure 3, the PP (18 questions) domain is analyzed, with 5 innovative questions achieving a DI of ≥0.20, demonstrating its effectiveness in assessing the examinees’ clinical skillset.

**Figure 4:**
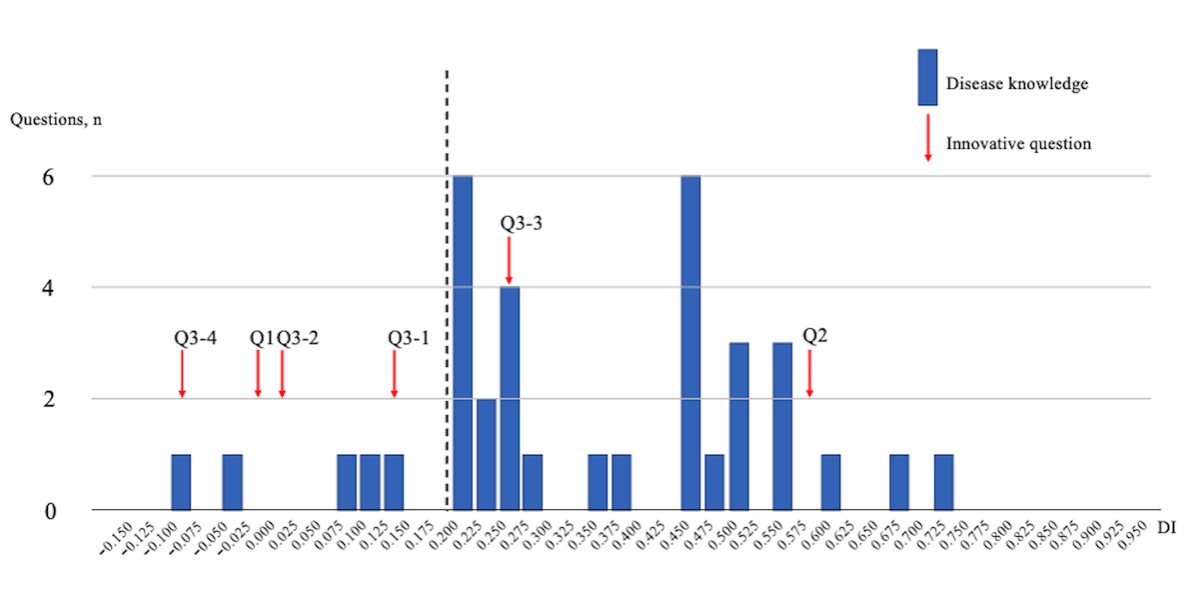
DIs of the examination scores of the General Medicine In-Training Examination: disease knowledge (36 questions). DI: discrimination index; Q: question.

Finally, Figure 4 presents the DI for the DK (36 questions) domain, with 2 innovative questions achieving a DI of ≥0.20, reflecting its potential as a moderate discriminator of examinees’ understanding.

These figures collectively underscore the CSV innovative examination’s capacity to gauge clinical competence effectively, with each domain’s innovative question serving as a significant indicator of the examinees’ capabilities. In particular, for the innovative question Q2, a DI of ≥0.20 was found for both the total score and all 4 domains, indicating its robustness in differentiating examinee proficiency.

A total of 23 (41%) participants answered Q4, the anonymous questionnaire to assess the participants’ views on the CSV innovative examination. Regarding whether the simulated patient examination video was better suited to assessing their clinical competence than the traditional all-text format (Q4-1), 12 (52%) participants answered positively, 4 (17%) answered negatively, and 7 (30%) provided a neutral response. Regarding whether the video simulation was realistic enough for them to assess the patient (Q4-2), 18 (78%) responded affirmatively. Regarding whether the experience increased their motivation to learn, 17 (74%) responded positively.

**Table 1:** Discrimination index^a^.

Domain (questions, n)	Question 1	Question 2	Question 3-1	Question 3-2	Question 3-3	Question 3-4
Medical interview or professionalism (8)	0.48	0.38	0.94	0.74	0.30	0.61
Clinical diagnosis (18)	0.50	0.40	0.77	0.56	0.27	0.18
Physical examination or procedure (18)	0.52	0.35	0.39	0.19	0.22	0.39
Disease knowledge (36)	–0.09	0.58	0.13	0.04	0.27	–0.10
Total (80)	0.06	0.47	0.10	–0.06	0.01	–0.12
Question type	MC^b^	FD^c^	FD	FD	FD	FD

^a^A discrimination index of ≥0.20 indicates that the question had high discriminatory power; a discrimination index of >0.40 indicates that the question was a very good measure of the participant’s qualifications.

^b^MC: multiple choice.

^c^FD: free description (<100 words).

## Discussion

### Principal Findings

Residency is the final stage of medical education and supervised clinical practice. The traditional all-text GM‑ITE was designed to elicit practical feedback on the preresidency training to identify areas of improvement by objectively assessing residents’ clinical knowledge in 4 areas: MP, CD, PP, and DK. Medical education has historically relied on MCQs to assess learning [20,21]. However, some studies have explored “context-rich” MCQs that embed test items in a clinical vignette [22,23]. This study delved beyond a written clinical vignette by creating a video simulation of a patient examination in an emergency room. The strength of ratings regarding the measures of different components of clinical reasoning indicates that although MCQs are effective in leading or working diagnosis and management and treatment, they are weak in hypothesis generation, problem representation, and diagnostic justification [7]. Conversely, it has been found that while differential diagnosis, leading or working diagnosis, diagnostic justification, and management and treatment are effective in essay style (free text), they are relatively weak in information gathering [24]. This finding suggests that CSV-based test modules could provide a more accurate measure of participants’ clinical knowledge and abilities than the GM-ITE.

Education, including medical education, has increasingly embraced computer-based testing. Today, students are accustomed to answering questions and writing essays via computer-based testing. This study designed a single video simulation to assess the knowledge and skills of residents from the nonverbal information portion of the national medical licensing examination domains, particularly general theory. We included information from 3 domains in a single question, and the participants obtained high scores. This finding suggests that a single CSV module could test multiple skills and knowledge areas of residents. In other words, using innovative CSV-based questions could provide more realistic assessments while making the examinations more efficient.

The 3 domains covered in the CSV innovative examination Q1 (MP, CD, and PP) indicated DI of 0.4 or higher; the GM-ITE means were 0.32 (SD 0.13), 0.32 (SD 0.16), and 0.31 (SD 0.18), respectively. Therefore, the successful participants (based on GM-ITE scores) had higher scores on these domains in the CSV innovative examination question than in the GM-ITE. Q1 required participants to select 3 options from the MCQ (2 cutoffs per question). We found that the CSV could cover 3 separate domains in a single MCQ.

CSV innovative examination Q2 required a descriptive response; specifically, the participants needed to name the most likely diagnosis. Two physicians (KS and SF) independently assessed the diagnoses and achieved an agreement rate of 1.00. The DI of Q2 was 0.4 or higher for symptomatology or clinical reasoning and diseases and 0.3 or higher for general theory, physical examination, and clinical techniques. The overall GM-ITE scores had a high identification index of 0.47. Specifically, the CSV innovative examination Q2’s requirement for participants to provide a definitive diagnosis allowed for a comprehensive assessment across all domains included in the GM-ITE. Furthermore, Q2 was distinguished as the sole question that demonstrated high DIs across individual disease categories. In addition, Q2 was the only question that also presented a high DI in each disease category.

CSV innovative examination Q3 required participants to provide an SBAR (situation, background, assessment, and recommendation) report using a total of 400 words or fewer. Two physicians (KS and SF) scored the responses independently and then rated each response against the scoring criteria and added them together. The agreement rate was as high as 0.92. It was observed that Q3 lowered the overall DI score to a high level in the general discussion. In other words, Q3 was easier for all the participants to answer than the other questions. For Q3-1 and Q3-2, the high discriminative ability was lowered for symptomatology and clinical reasoning. However, for each theory of disease, all the DIs were low, with some negative results. Therefore, most participants were better able to describe the patient’s situation and background than provide an assessment and recommendations.

This study is significant in that it provided “content-rich” clinical information. In addition to obtaining all the information normally provided in the conventional paper–based examinations, the participants had the advantage of seeing and hearing the various symptoms portrayed by a professional actor. In addition, medical interviews with patients and their families can reveal useful nonverbal information such as tachypnea and expressions indicating anxiety and pain levels. Gathering clinical information through diagnostic inference is critical in real-life scenarios. Participants may have performed better in certain domains covered in Q1-Q3 compared to their GM-ITE scores for the same domains owing to the CSV’s heightened sense of immediacy (seeing “real” people rather than reading about them) and the opportunity for diagnostic inferences in workplace-based assessments. This finding may indicate a development of clinical competence from the level of “knows how” to “shows” in Miller’s pyramid, which could lead to an advanced assessment in the cognitive domain.

### Comparison to Prior Work

The discriminative efficacy of the CSV’s innovative examination in this study aligns with similar interventions. A study comparing simulation and video-based training for acute asthma management found that both methods significantly improved MCQ posttest scores, indicating an enhanced understanding of clinical methods [25]. Additionally, a study conducted at a university hospital in Pakistan revealed that a hybrid model combining video-based learning with simulation increased students’ confidence and performance in clinical skills. This suggests that digital and multimedia-enhanced methods may surpass traditional teaching modalities in certain aspects of medical education [26]. These comparisons underscore the potential of CSV-based assessments to provide a more nuanced and comprehensive measure of clinical competencies, potentially bridging theoretical knowledge and practical application more effectively in medical training.

### Limitations

Although this study reveals important findings, it has several limitations. First, the number of participants included in the study was low. For the data to be more valid, the number of examinees needs to be increased. However, adding more participants would also increase the test-scoring burden, which calls the viability of CSV-based testing into question. In this study, 2 physicians (KS and SF) scored the written questions. Increasing the number of examinees would also increase the time and effort required to score the results. If all of the approximately 8000 examinees who took the GM-ITE completed the CSV innovative examination module, the scoring time required would be untenable, and adding more CSV-based modules would compound the problem. One way to overcome this limitation could be the use of a morphological analysis or to only score a statistically significant sampling.

Another limitation is related to the authenticity of the CSV. We created the abnormalities in the “patient,” such as the heart murmur and loud P2, by synthesizing sounds. We could not represent some aspects, such as the enhancement of systolic murmur on inspiration, and the apex beat was not clear, which might have confused the examinees. Furthermore, the time and expense involved in creating high-quality, realistic clinical cases would likely reduce the number of modules that could be used, which might enable the test takers to gain prior knowledge of the “correct” answers, therefore defeating the purpose of the test. Future research should determine the feasibility of including real cases and patients to maximize verisimilitude and reduce personnel and production expenses.

### Conclusions

The findings of this study suggest that the CSV showed a high identification index for overall and multiple domains of competence in the conventional GM-ITE. The participants liked being able to “examine” the patient and receive visual and auditory clinical information, which improved their test scores. Overall, the findings showed that CSV modules simulating real-world clinical examinations assessed residents’ clinical competence successfully in multiple domains.

## Appendix

Multimedia Appendix 1 Innovative examination.

## Abbreviations

CD: clinical diagnosis
CSV: clinical simulation video
DI: discrimination index
DK: disease knowledge
GM-ITE: General Medicine In-Training Examination
JAMEP: Japan Institute for Advancement of Medical Education Program
MCQ: multiple-choice question
MP: medical interview or professionalism
PGY: postgraduate year
PP: physical examination or procedure
SBAR: situation, background, assessment, and recommendation

## Data Availability

The data sets generated during and analyzed during this study are available from the corresponding author on reasonable request.
